# Anti-lipid phosphate phosphohydrolase-3 (LPP3) antibody inhibits bFGF- and VEGF-induced capillary morphogenesis of endothelial cells

**DOI:** 10.1186/1478-811X-3-9

**Published:** 2005-08-02

**Authors:** Kishore K Wary, Joseph O Humtsoe

**Affiliations:** 1 Institute of Biosciences and Technology, Texas A&M University System Health Science Center, Texas Medical Center, 2121 W. Holcombe Blvd., Houston TX-77030, USA; 2 Department of Cell and Tissue Biology, University of California San Francisco, 521 Parnassus Ave., CA-94143, USA

**Keywords:** bFGF, capillary morphogenesis, collagen matrices, endothelial cells, VCIP, VEGF

## Abstract

**Background:**

Angiogenesis, or the remodeling of existing vasculature serves as a lifeline to nourish developing embryos and starved tissues, and to accelerate wound healing, diabetic retinopathy, and tumor progression. Recent studies indicate that angiogenesis requires growth factor activity as well as cell adhesion events mediated by α_5_β_1 _and α_v_β_3 _integrins. We previously demonstrated that human lipid phosphate phosphohydrolase-3 (LPP3) acts as a cell-associated ligand for α_5_β_1 _and α_v_β_3 _integrins. Here, we test the hypothesis that an anti-LPP3 antibody can inhibit basic fibroblast growth factor (bFGF)-and vascular endothelial growth factor (VEGF)-induced capillary morphogenesis of endothelial cells (ECs).

**Results:**

We report that bFGF and VEGF up-regulate LPP3 protein expression in ECs. Immunoprecipitation analyses show that LPP3 is a cell surface protein and undergoes N-glycosylation. Fluorescent activated cell sorting (FACS) data suggest that anti-LPP3-RGD detects native neoepitope on the surface of activated ECs. Moreover, we demonstrate LPP3 protein expression in tumor endothelium alongside VEGF. The embedding of ECs into three-dimensional type I collagen in the presence of bFGF and VEGF induce capillary formation. Importantly, we show that the addition of an anti-LPP3 antibody specifically and significantly blocks bFGF- and VEGF-induced capillary morphogenesis of ECs.

**Conclusion:**

These data suggest that activated ECs as well as tumor endothelium express LPP3 protein. In an *in vitro *assay, the anti-LPP3-RGD specifically blocks bFGF and VEGF induced capillary morphogenesis of ECs. Our results, therefore, suggest a role for LPP3 in angiogenesis.

## Background

Angiogenesis, the sprouting or remodeling of preexisting quiescent blood vessels, is critical for embryonic development, wound healing, and various pathological conditions such as tumor progression, complications associated with acquired immune deficiency syndrome (AIDS), rheumatoid arthritis, and diabetic retinopathy [1-4]. Angiogenesis can be initiated by hypoxic tumors, inflammation or an increased accumulation of pro-angiogenic factors. These factors, in turn, trigger secretion of matrix metalloproteinases (MMPs) that dissolve the basement membrane. This MMP-mediated membrane dissolution is an essential event for subsequent EC activation, migration, and capillary formation [1-6]. Angiogenesis is regulated through a dynamic balance between pro- and anti-angiogenic factors [1-4]. Angiogenic mediators include growth factors such as basic fibroblast growth factor (bFGF), vascular endothelial growth factor (VEGF), collagen and fibronectin, and proteases such as MMPs [2,4,6-8]. VEGF signaling activates ECs through VEGF receptor-1 (VEGFR-1, also known as Flt) and VEGFR2 (KDR/Flk-1) tyrosine kinase receptors, and promotes cell migration, survival, proliferation and differentiation [5,6,9]. The microenvironment surrounding a tumor is generally rich in VEGF, which is upregulated in response to hypoxia and can directly activate ECs to initiate tumor angiogenesis, growth and metastatic deposits [1-4,9]. Both bFGF and VEGF are able to induce tumor angiogenesis and wound healing, as well as contribute to unwanted angiogenesis [2,4-6,9]. Addition of bFGF and VEGF can increase the expression of EC integrins, a family of cell surface receptors that regulate cell adhesion events [2,10-13]. In particular, α_5_β_1 _and α_v_β_3 _integrins mediate adhesion, migration, and proliferation of endothelial cells by interacting with extracellular matrix (ECM) proteins such as fibronectin, fibrin, and vitronectin [13-15]. In addition, integrins also mediate cell-cell interactions by associating with counter-receptors or cell associated integrin ligands [16,17]; such interactions generate both chemical and mechanical signals that influence cellular behavior [18-22].

Our ability to target neo-epitopes expressed by tumor-endothelium could potentially minimize the toxicity and drug-resistance associated with conventional chemotherapy treatment of solid tumors [9]. Recently, we identified lipid phosphate phosphohydrolase-3 (LPP3), also called phosphatidic acid phosphatase-2b (PAP2b), VEGF and type I collagen inducible protein (VCIP) in a functional assay of angiogenesis [23,24]. Lipid phosphate phosphohydrolases (LPPs) dephosphorylate polar lipid signaling molecules, both within and outside cells [25-27]. Structurally, all LPPs display a 6-transmembrane channel-like organization [29-32]. Both the N-and C-terminal segments are located in the cytoplasm [32,33]. There are three extracellular loops, and the proposed 2^nd ^extracellular loop of LPP3 contains a lipid phosphatase, one cell-adhesion motif, and a N-glycosylation site [23,29,32,33]. LPP3 protein has been identified within intracellular organelles as well as on the cell surface, and in both locations it exhibits ectoenzyme activity [29-32]. Previously we have shown that LPP3-RGD (RGE in mice) can act as a cell-associated integrin ligand and mediate cell-cell interactions [23,28]. Consistent with our findings, confocal image analyses demonstrated that green fluorescent protein-LPPl remains apically sorted, whereas green fluorescent protein-LPP3 co-localized with E-cadherin in cell-cell junctions and the basolateral domains of polarized MDCK cells [23,33]. Transfection of mutants as well as swapping experiments have established that LPP1 protein contains an apical targeting signal sequence (FDKTRL) in its N-terminal segment; in contrast, LPP3 protein contains dityrosine (109Y/110Y) cell-cell and basolateral sorting motifs [33]. Unlike *Lpp2*, whose function is dispensable for embryonic development, *Lpp3 *is required for extra-embryonic vasculogenesis and axis patterning [34,35], raising the possibility that the function of the LPP3 protein may also be to mediate adult, as well as pathological, angiogenesis.

We previously showed that anti-LPP3-RGD blocks cell aggregation (cell-cell interactions) that is mediated by α_5_β_1 _and α_v_β_3 _integrins [23]. In the current study, we examine whether an anti-LPP3-RGD antibody can inhibit bFGF- and VEGF-mediated capillary morphogenesis of ECs. In this study, we demonstrate that the addition of bFGF and VEGF angiogenic cytokines stimulate the expression of LPP3 protein of ECs. We further show that tumor endothelium express LPP3 protein. By embedding ECs in a three-dimensional type I collagen matrix followed by treatment with bFGF and VEGF to induce formation of capillaries, we demonstrate the ability of anti-LPP3 antibodies to inhibit bFGF- and VEGF-induced capillary morphogenesis. These findings are the first to our knowledge to suggest a mechanism by which anti-LPP3-RGD antibodies may inhibit capillary morphogenesis of ECs.

## Results

### Basic FGF and VEGF induce expression of LPP3 in HUVECs

**Figure 1:**
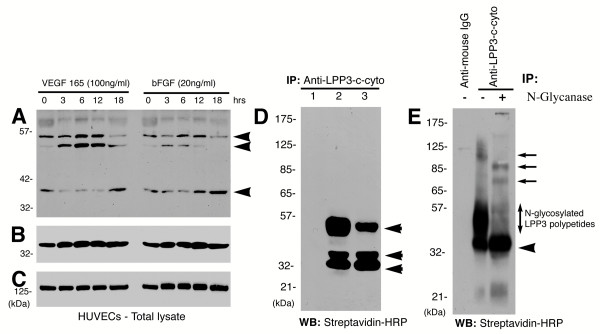
**Expression of LPP3 protein in monolayer ECs. **Confluent ECs (passage 4) were starved for 6 h in conditioned medium (M199 supplemented with 0.2% BSA + 1× ITS), and then stimulated with VEGF^165 ^(100 ng/ml) or bFGF (20 ng/ml) for various durations, as indicated. Cells were solubilized, clarified by centrifugation, and the protein concentrations were determined. Samples were subjected to SDS-PAGE and analyzed by immunoblotting with: **(A) **Rabbit anti-LPP3-c-cyto polyclonal antibody (2.0 μg/ml). Anti-LPP3-c-cyto antibody detects unprocessed LPP3 protein that appears as ~36 kDa, and two major polypeptides migrate below the ~52 kDa molecular weight marker, LPP3 polypeptides are indicated by arrowheads; **(B) **Anti-PCNA monoclonal antibody (0.5 μg/ml). **(C) **Anti-Fak monoclonal antibody (1.0 μg/ml). **(D) Cell surface biotinylation of intact cells and immunoprecipitation analysis. **K562 (lane 1, unstimulated), HUVECs (lane 2, stimulated with VEGF, 100 ng/ml 6 hours), and HUVECs (lane 3, stimulated with bFGF, 20 ng/ml for 6 h) subjected to cell surface biotinylation, lysed in RIPA buffer, clarified by centrifugation and immunoprecipitated using anti-LPP3-c-cyto (5 μg) antibody. Immunocomplexes were resolved by 10% SDS-PAGE under reducing condition and analyzed by ligand blotting with streptavidin-HRP (1:10000). Data shown are representative of those obtained in at least three separate experiments, with similar results. **(E) De-N-glycosylation of LPP3 protein. **Monolayer HUVECs (passage 4) were stimulated with VEGF^165 ^for 6 h and subjected to cell surface biotinylation, lysed in RIPA buffer, clarified by centrifugation, and immunoprecipitated by either rabbit IgG (control) or anti-LPP3-c-cyto antibodies, as indicated. Following immunoprecipitation with anti-LPP3-c-cyto antibodies, the contents were equally divided into two tubes. One tube was left untreated, and the second tube treated with N-Glyganase (50 units of PNGaseF) enzyme at 37°C for 3 h. Immunocomplexes were analyzed by ligand blotting with streptavidin-HRP (1:10000). Arrowheads indicate LPP3 polypeptides. Arrows indicate unknown polypeptides. Molecular weights are given in kiloDaltons (kDa). Data shown are representative of those obtained in at least three separate experiments, with similar results.

Hypoxic tumors *in vivo *and many cell lines *in vitro *secrete bFGF and VEGF. Both bFGF and VEGF are components of the tumor microenvironment capable of activating ECs. To evaluate the potential role of LPP3 in angiogenesis, we investigated the effects of treatment of HUVECs with VEGF and bFGF. We stimulated monolayer HUVECs with either VEGF^165 ^or bFGF for various time periods between 0 and 18 h, and subjected lysates to Western blot analyses using an affinity purified anti-LPP3-cyto antibody (Fig. 1A). The expression of LPP3 protein levels was increased by >3-fold in response to VEGF^165 ^treatment (100 ng/ml) for 6 or 12 h (relative to control levels), whereas bFGF (20 ng/ml) had a significantly less robust effect on LPP3 levels during the same treatment duration (Fig. 1A). The concentrations of VEGF^165 ^and bFGF used in this experiment were optimal as evidenced by the observation that both factors activated extracellular-signal-regulated kinase (Erkl/2) in these cells in an independent experiment (data not shown). We observed that anti-LPP3-c-cyto antibody detects three major polypeptides, two of which (~52 and ~46 kDa) are slow and one (~36 kDa) that exhibits high mobility (Fig. 1A). Since, LPP3 contains a single consensus N-glycosylation site (170N) on the proposed 2^nd ^extracellular loop of the LPP3 protein, the high mobility anti-LPP3-c-cyto immunoreactive species is likely to be a post-translationally modified form. This idea is actually supported by our data with N-glycanase described elsewhere. As a positive control for the cytokines used, the membrane was stripped and re-blotted with an anti-proliferating cell nuclear antigen (PCNA) antibody (Fig. 1B). As expected, both cytokines increased PCNA expression to optimal levels. The level of Focal adhesion kinase (Fak) protein was measured to confirm equivalent protein loading (Fig. 1C).

Next, to determine the ability of anti-LPP3-c-cyto antibody to immunoprecipitate LLP3 antigen, we used ECs and human erythroleukemia (K562) cells (Fig. 1D). K562 cells that do not express LPP3 protein was included as a negative control. K562 cells were left unstimulated (Fig. 1D, lane 1), while ECs were stimulated with VEGF (Fig. 1D, lane 2) and bFGF (Fig. 1D, lane 3), and subsequently subjected to cell surface biotinylation and immunoprecipitation with an anti-LPP3-c-cyto antibody and analyzed by ligand blotting with Streptavidin-HRP (Fig. 1D). We found that the anti-LPP3-c-cyto did not immunoprecipitate 36–52 kDa polypeptides from K562 cells (negative control cell line); in contrast, ligand blotting with streptavidin-HRP detected three major polypeptides (36, 42 and 52 kDa, indicated by arrows) (Fig. 1D). These data suggest that the LPP3 antigen is exposed on the extracellular surface of ECs and can be immunoprecipitated by an anti-LPP3-c-cyto antibody.

LPP3 contains a single N-glycosylation site (170N, accession number O14495) [23]. Several pilot experiments suggested that, depending upon how cells are cultured, the variation in the extent of LPP3 glycosylation can be complex and dramatic. To examine this possibility, we prepared cell extracts from ECs and subjected lysates to immunoprecipitation as indicated (Fig. 1E). After several washes with cell lysis buffer, immunoprecipitates were incubated with N-glycanase F (PNGaseF), an enzyme that cleaves the carbohydrate moiety. In doing so, we observed that most of the slow mobility (smeared) polypeptides disappeared, leaving behind a ~36 kDa unprocessed polypeptide (Fig. 1E). Consistent with previous reports, we observed that LPP3 is N-glycosylated [30,31], and the extent of N-glycosylation appears to be cell type- and culture condition-dependent.

### The LPP3 is a cell surface antigen

**Figure 2:**
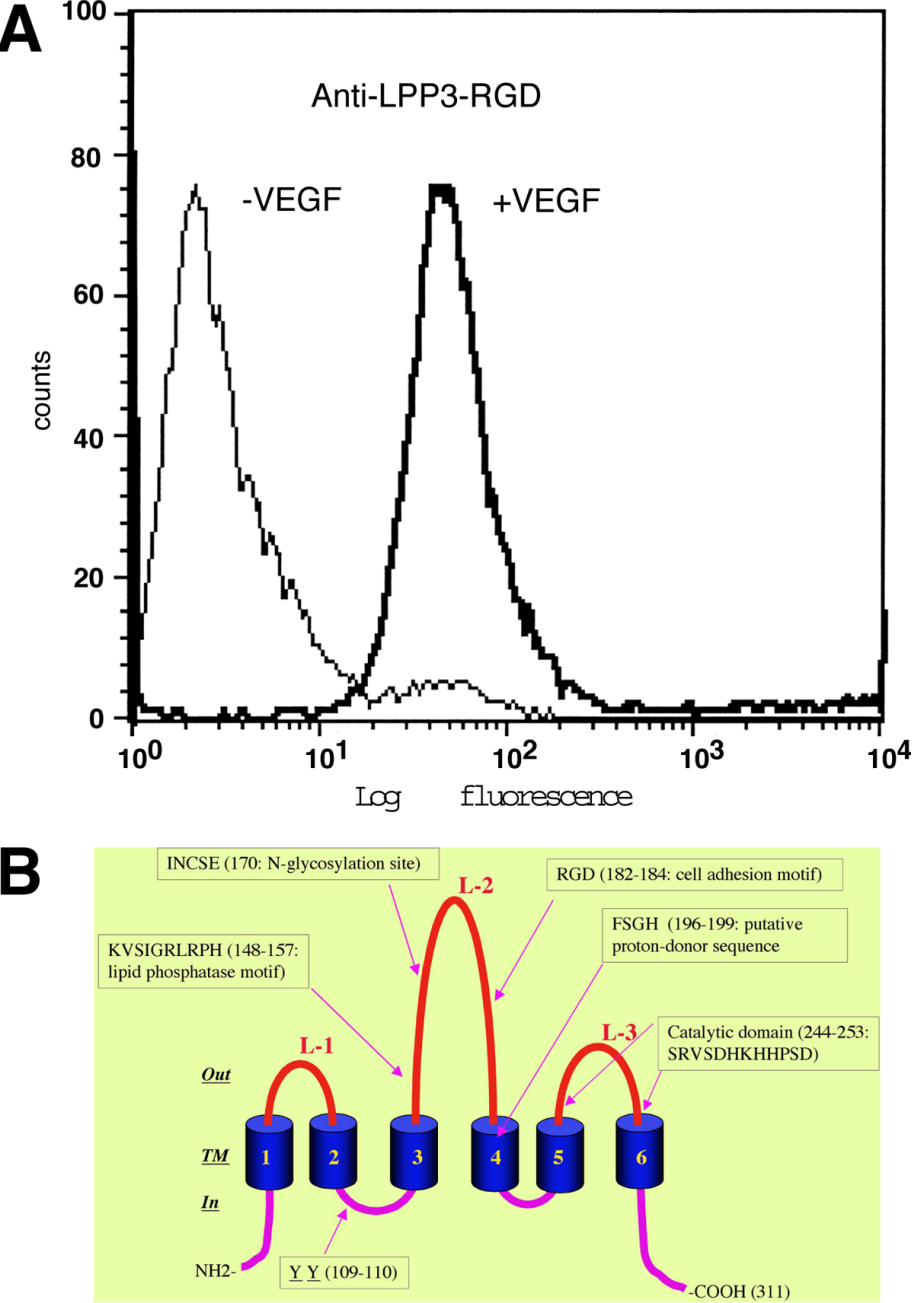
**LPP3 is a cell surface antigen. A) **HUVECs (passage 4) were serum-starved for 6 h, thereafter, left untreated (-) or treated with VEGF^165 ^(100 ng/ml) for 6 h and subjected to fluorescent activated cell sorting (FACS) using indicated antibodies. **B) **Schematic representation of human LPP3 protein showing 6-transmembrane organization. Proposed 3 extracellular loops (L-l, -2, and -3) are as shown. One lipid phosphatase motif, a cell adhesion sequence, a putative proton donor sequence and a dityrosine basolateral targeting sequence are as shown. Both N-and C-terminals of LPP3 protein are located inside the cytoplasm.

Anti-LPP3-RGD antibody was raised by injecting rabbit with a synthetic peptide modeled after the proposed 2^nd ^extracellular loop of LPP3 protein (EGYIQNYRCRGDDSKVQEAR) [23]. Previously we relied on the specificity of the anti-LPP3-RGD antibody to analyze tumor sections and inhibit LPP3-mediated cell-cell interactions [23]. In the current study, we determined the ability of anti-LPP3-RGD to detect native LPP3 antigen of intact ECs. Towards this end, ECs were either left unstimulated or were stimulated with VEGF, incubated with anti-LPP3-RGD and subjected to fluorescence activated cell sorting (FACS). In contrast to unstimulated ECs that do not express LPP3 protein significantly, the addition of VEGF induced cell surface expression of LPP3 protein of monolayer ECs (Fig. 2A). This result indicates that VEGF stimulates expression of LPP3 antigen on the surface of ECs, which can be detected by anti-LPP3-RGD antibody. This data also suggests that anti-LPP3-RGD antibody detects intact and native LPP3 antigen neoepitope expressed by activated ECs. A schematic diagram showing topological and structural organization of LPP3 is shown (Fig. 2B).

### Tumor endothelium expresses LPP3 protein

**Figure 3:**
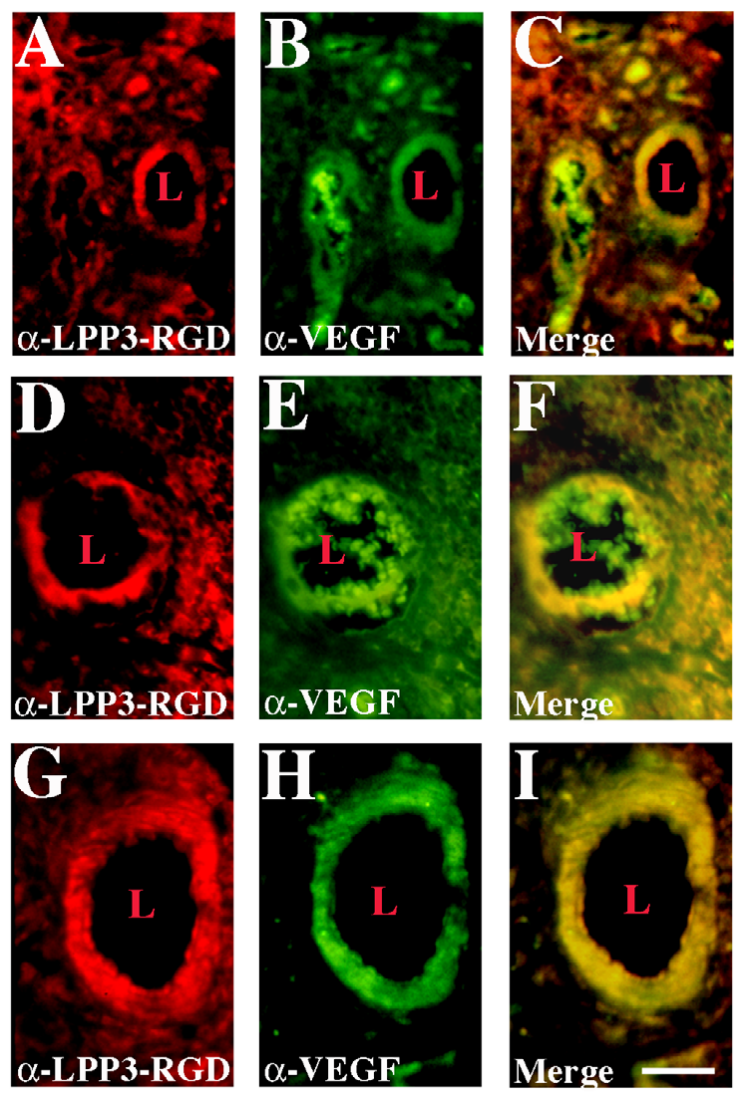
**Tumor endothelium express LPP3 protein alongside VEGF. **Paraffin-embedded angioma **(A-C) **and hemangioma **(D-I) **tumor tissue sections (4 μm) were subjected to antigen retrieval, and sequentially incubated with the indicated antibodies. After washing with PBS, sections were incubated with donkey anti-goat/rabbit IgG conjugated to Texas-red (red) and goat anti-mouse IgG conjugated to FITC (green). C, F, and I images represent overlays of A, B; D, E; and G, H respectively. Images were captured below saturation level. Merged yellow represents co-expression. Data shown are representative of those obtained in at least three separate experiments, with similar results. (L, lumen; Magnification, 100×; Bars, 50 μM).

It is apparently clear that the tumor microenvironment contains bFGF and VEGF. Because bFGF and VEGF induce LPP3 expression in cultured ECs, we hypothesized that LPP3 protein might be similarly expressed by tumor-endothelium. To examine this hypothesis, serial angioma and hemangioma sections were subjected to immunostaining with anti-Flk-1, anti-PECAM-1 (CD31), and von Willebrand factor (vWF) to establish the presence of ECs and blood vessels as previously described [23]. We have previously shown that quiescent skin blood vessels are negative for anti-LPP3-RGD immunoreactivity [please see reference 23 for online supplemental data]. Hypoxic tissues as well as inflamed tissues are known to express VEGF [1,4]. Consistent with these reports, our data indicate that VEGF (green) is diffusedly distributed throughout angioma and hemangioma tissues, with significantly higher expression in the blood vessels (Fig. 3B,3E,3H). As shown in Fig. 3A,3D and 3G, LPP3 (red) protein appears to co-localize with VEGF in the angioma, including in the endothelium where the yellow ring like structure indicates colocalization; however, their molecular proximity remains unknown. Similarly, the expression of LPP3 protein was coincident with VEGF expression in the hemangioma section examined (Fig. 3G,3H,3I). These data demonstrate that both LPP3 and VEGF are diffusedly distributed within tumor vasculature, and LPP3 expression may not be exclusively restricted to ECs. Incubation of the antibodies with peptides that had been used to generate the primary antibody blocked immunoreactivity, confirming the specificity of antibodies used, as previously described [23]. This data is consistent with our earlier observation that the LPP3 protein is highly expressed in tumor endothelium [23].

### Inhibition of bFGF and VEGF induced capillaries by anti-LPP3-RGD antibodies

A considerable number of studies indicate that bFGF- and VEGF-mediated signaling, as well as cell adhesion events mediated by α_5_β_1 _and α_v_β_3 _integrins, critically determine the outcome of angiogenesis. Because the LPP3-RGD protein acts as a cell-associated integrin ligand for α_5_β_1 _and α_v_β_3 _integrins, we hypothesized that an anti-LPP3-RGD antibody could inhibit cell-cell interactions that may impede the ability of ECs to undergo capillary morphogenesis (also called tubulogenesis). To evaluate the capacity of anti-LPP3-RGD to block capillary morphogenesis of ECs, we employed early passage (between 3–4 total) ECs. Typically, we embed ECs between two layers of type I collagen matrices, and treat them with bFGF and VEGF in the presence of 20% serum. As previously described, the process of capillary formation by ECs in a three-dimensional type I collagen take place over a period of 24 to 72 h, and requires the addition of bFGF and VEGF [23,24].

For the purpose of this study, capillaries formed after 24 h of culture in 3D collagen were considered "pre-formed vessels", whereas capillaries formed after more than 24 h of culture were considered "new capillaries". We used affinity purified rabbit anti-IgG (control) polyclonal antibodies (pAbs) and mouse anti-MHC class II (W6/32) monoclonal Abs (mAbs), and mouse anti-β_1 _(4B4) and anti-α_v_β_3 _(LM609) mAbs, as negative and positive controls, respectively.

**Figure 4:**
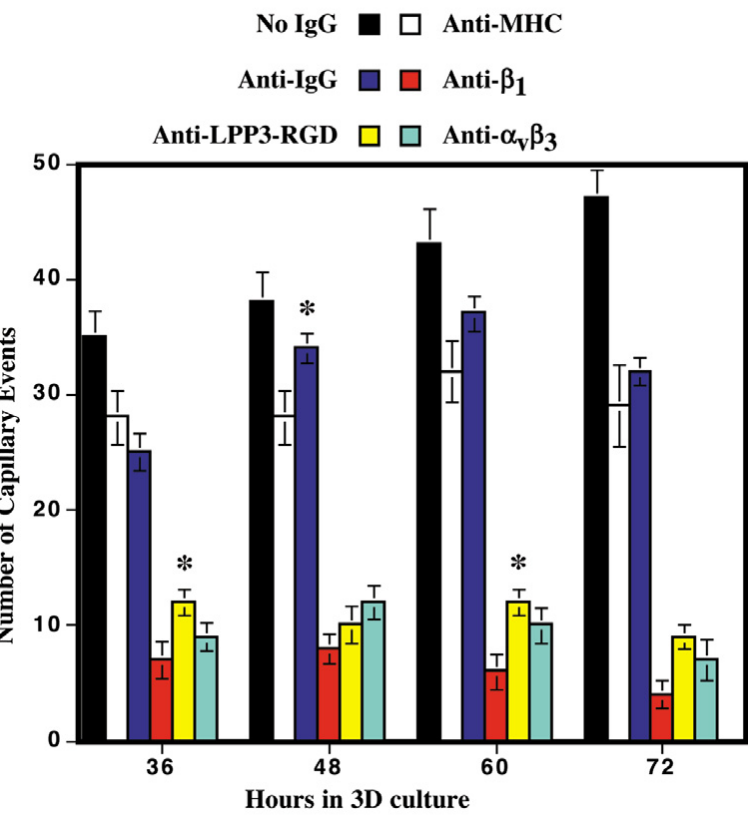
**Effect of specific antibodies on pre-formed capillaries. **ECs were cultured in 3D collagen matrices in the presence of bFGF and VEGF^165^. Cultures were treated with the indicated antibodies at 24 h and processed at various time points (indicated). The 3D cultures were fixed, serial sections prepared and stained with eosin. The capillaries were counted as described in methods section. Values represent the mean ± SEM obtained from three independent experiments that used five wells in each case. * *P *< 0.02.

**Figure 5:**
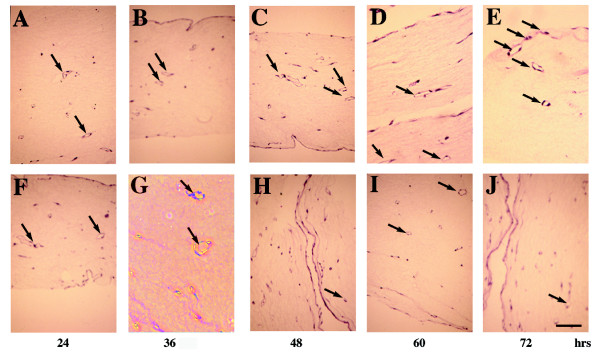
**Inhibition of bFGF and VEGF induced capillary morphogenesis of ECs in 3D type I collagen matrix. **The samples shown in the upper panels **(A-E) **were treated with anti-MHC class II mAbs, whereas those in the lower panels **(F-J) **with anti-α_v_β_3 _integrin antibodies. Cross sections were stained with acidified eosin as described in methods. Magnification, 100×. Bar, 200 μM.

In the absence of antibodies, we observed an increased number of capillaries formed from 36 to 72 h in culture (Fig. 4, filled black bar). The addition of an anti-MHC mAb (empty bar) and rabbit IgG (filled blue bar) resulted in minimal inhibition, and for the purpose of our study we consider these minimal inhibitions as baseline (Fig. 4). In contrast, the addition of the anti-β_1 _(filled red bar) and anti-α_v_β_3 _(filled green) mAbs caused regression of the pre-formed capillaries (Fig. 4). Anti-α_v_β_3 _mAbs reduced the number of pre-formed capillaries by more than 50%, suggesting that other cell surface proteins, such as fibronectin-binding integrin α_5_β_1 _and collagen/laminin binding integrins may also mediate capillary morphogenesis. Indeed, anti-β_1 _integrin subunit mAbs inhibited pre-formed interconnections and reduced the number of capillaries by ~60–70% (Fig. 4). No two mAbs were added simultaneously since it has been reported that α_5_β_1 _and α_v_β_3 _integrin antibodies together induce complete collapse and regression of tubules *in vitro *[37,38]. Unexpectedly, the addition of the anti-LPP3-RGD antibody (filled yellow bar) had a dramatic effect on bFGF and VEGF-induced capillary formation (Fig. 4). The effect of anti-LPP3-RGD was comparable to anti-α_v_β_3 _mAbs (Fig. 4, solid yellow bar). Representative cross sections of 3D gel are shown (Fig. 5). Arrows indicate lumen like structures. These data suggest that antibodies affecting cell adhesion, migration and cell-cell interaction events inhibit capillary morphogenesis by ECs *in vitro*. These data demonstrate that an affinity-purified rabbit anti-LPP3-RGD polyclonal antibody can inhibit bFGF- and VEGF-induced capillary formation *in vitro*.

## Discussion

We previously identified LPP3 in a functional assay of angiogenesis and reported that human LPP3 protein mediates cell-cell interactions [23,24]. Although the proposed cell adhesion sequences of human (RGD) and mouse (RGE) LPP3 are not identical, we observed that, in response to long-term cell adhesion, both sequences efficiently ligate α_5_β_1 _and α_v_β_3 _integrins, and these adhesion events do not require protein synthesis [28]. Many previous studies have described a correlation between over-expression of LPP3 as an enzyme and down-regulation of cell signaling. However, the critical importance of *Lpp3 *gene function during developmental angiogenesis is underscored by the observation that mice embryos lacking *Lpp3 *die at day E7.5 due to a dearth of functional vasculature [35]. This report suggested that LPP3 protein regulates cellular interactions [35]. It is clear that further investigation will be necessary to elucidate the function of LPP3. Regardless of the mechanism, LPP3 is likely to be required during both adult and pathological angiogenesis. Therefore, we hypothesize that inhibition of LPP3 protein function blocks angiogenesis. Here, we asked the question whether an anti-LPP3-RGD antibody inhibits bFGF and VEGF induced capillary morphogenesis of ECs. Indeed, we found evidence that an anti-LPP3-RGD antibody can inhibit capillary morphogenesis of ECs.

It has become increasingly clear that bFGF- and VEGF-induced angiogenesis requires integrin-mediated adhesion events, a process by which ECs maintain cell-cell contact, survive, migrate, and proliferate [11-14,17]. ECs are known to express α_2_β_1, _α_3_β_1, _α_5_β_1, _α_6_β_1, _α_v_β_3 _and α_v_β_5_, integrins [13,17,18]. Several integrins have been suggested to play a role in each step of these processes; of these, α_5_β_1 _and α_v_β_3 _integrins appear to be key players [11-14]. Previously, we provided evidence that a subset of integrins, including α_1_β_1, _α_5_β_1 _and α_v_β_3 _are able to promote the EC cell cycle progression through the Shc pathway [17,18,21]. *In vitro *and *in vivo *assays have provided evidence that interference with the function of α_5_β_1 _and α_v_β_3 _integrins block bFGF- and VEGF-induced angiogenesis, suggesting that α_5_β_1_- and α_v_β_3_-mediated signaling networks cooperate by regulating a similar angiogenic signaling cascade [39-42]. Because signaling from α_5_β_1 _and α_v_β_3 _integrins are critical for EC functioning, perhaps depriving LPP3-mediated cell-cell interactions interrupts several important signaling pathways, thereby inducing regression of capillary morphogenesis by ECs.

Our data indicate that pathophysiologically relevant agonists, e.g. bFGF and VEGF cytokines, can stimulate the expression of LPP3 protein in ECs. These data imply that LPP3 protein expression is likely to be associated with inflamed tissues and organs that require angiogenesis. We also demonstrate that LPP3 protein is up-regulated in tumor endothelium. We also show the ability of anti-LPP3-RGD to detect native LPP3 antigen on the cell surface of ECs. Considering the ability of *Lpp3 *to regulate embryonic vasculogenesis, and to ligate α_5_β_1 _and α_v_β_3 _integrins, we propose that the function of LPP3 protein is pro-angiogenic. Accordingly, we show that specific inhibition of LPP3 with an anti-LPP3-RGD polyclonal antibody inhibit bFGF- and VEGF-induced capillary morphogenesis. Currently, a function blocking anti-LPP3 monoclonal antibody is not available. It is clear that further studies would be necessary to test the ability and efficiency of various anti-LPP3 antibodies and peptides to inhibit angiogenesis *in vivo*.

## Methods

### Cells and Reagents

Human umbilical vein endothelial cells (HUVECs) were purchased from Cambrex Bio Science Inc. (Walkersville, MD). Media 199, antibiotic solution, L-glutamine and all other cell culture reagents were purchased from InVitrogen Corp. (Carlsbad, CA). Recombinant human vascular endothelial growth factor (VEGF^165^), basic fibroblast growth factor (bFGF), and anti-VEGF (MAB293) were purchased from R&D Systems Inc. (Minneapolis, MN); adult human serum-AB from Gemini Bioproducts (Woodland, CA); bovine skin-derived type I collagen from Cohesion Technologies, Inc. (Palo Alto, CA). Affinity-purified anti-α_v_β_3 _integrin (LM609), anti-PCNA and anti-VE-cadherin (MAB1989) monoclonal antibodies (mAbs) were purchased from Chemicon International Inc. (Temecula, CA) and anti-KDR/Flk-1 (sc-6251) mAb from Santa Cruz Biotechnology Inc. (Santa Cruz, CA). Mouse anti-Fak monoclonal antibody was purchased from Upstate Biotechnology Inc. (Lake Placid, NY); anti-human β_1 _integrin subunit (clone 4B4) mAbs from Beckman Coulter Inc. (Fullerton, CA); and anti-MHC class II (W6/32) from Sigma Chemical Com., (St. Louis, MO). The preparation of rabbit anti-LPP3-RGD (previously called anti-VCIP-RGD or anti-PAP2b-RGD) and anti-LPP3-c-cyto (previously called anti-VCIP-c-cyto or anti-PAP2b-c-cyto) polyclonal antibodies (pAbs) has been previously described [23,24].

### De-glycosylation of LPP3 protein

Monolayer ECs (2 × 10^7^) at passage 4 were stimulated with VEGF^165 ^and subjected to cell surface biotinylation as previously described [23,24]. Biotinylated ECs were solubilized in RIPA cell extraction buffer [50 mM HEPES, pH 7.5, 150 mM NaCl, 1.0% Triton X-100 (non-ionic), 0.25% SDS (anionic), 0.25% sodium deoxycholate (anionic), and 2 mM EDTA, to which appropriate concentrations of proteases were added prior to use]. Extracts were clarified by centrifugation at 21,000 × g for 45 min at 4°C. Lysates were pre-absorbed at 4°C for 2 h by incubating with sepharose beads conjugated to rabbit IgGs. For each immunoprecipitation, approximately 1.5 mg total protein was used. Immunoprecipitation was carried out for 3 hr at 4°C. Immunocomplexes were washed five times with RIPA cell extraction buffer. For deglycosylation, immunoprecipitates were denatured in 1.0% SDS for 20 min at 90°C and washed twice with deglycosylation reaction buffer (New England Biolab., Beverly, MA). The deglycosylation (PNGaseF) reaction was initiated in a reaction volume of 100 μl containing 0.5% NP40 detergent and 50 units of PNGaseF enzyme at 37°C for 3 h. Samples were boiled in Lammeli reducing sample buffer and resolved by 10% SDS-PAGE and transferred to a nitrocellulose (NC) membrane. The NC membrane was blocked with 5% milk and 1% BSA in 1× TBS, 0.1% Tween and analyzed by incubating with streptavidin conjugated to horse-radish peroxidase (HRP) at a 1:10000 dilution. For FACS, monolayer ECs were starved for 6 h, thereafter, either left unstimulated or stimulated with VEGF (100 ng/ml) for 6 h. Cells were then non-enzymatically detached and subjected to FACS analyses as previously described [36].

### Monolayer and three-dimensional (3D) cell culture

Monolayer EC culture was performed as previously described [17,18,23,24]. The preparation of 3D collagen matrix has also been previously described [23,24]. Briefly, a viscous gel-like solution was prepared by mixing 7 ml of 3.0 mg/ml type I collagen solution with 1 ml of 10× Ml99 medium at 4°C, adjusting the pH to 7.5 with 0.1 N sodium hydroxide, adding 0.1 ml of 100× ITS, and adjusting to a final volume of 10 ml with sterile distilled water. The matrix was allowed to polymerize (solidify) for 30 min at 37°C. Next, unstarved proliferating ECs were gently resuspended (at 6 × 10^5 ^cells/ml in complete media), seeded onto solidified gels, and the dishes (24 well Coster cell culture dishes) were returned to a CO_2 _incubator for 2–3 h. At the end of 3 h, unattached cells were removed by gentle aspiration. Onto monolayer cells, a second layer of collagen gel was added and returned to a 37°C humidified CO_2 _incubator. Following solidification (~3 h), the matrix was layered with Ml99 medium containing 20% adult human serum-AB, 4 mM L-glutamine, 1× ITS, bFGF (20 ng/ml) and 100 ng/ml VEGF^165^. The old growth medium was removed and fresh medium was added every 24 h. Capillary formation was examined under a phase contrast microscope every 12 h.

### Inhibition Capillary Morphogenesis in 3D culture by anti-LPP3-RGD antibodies

ECs were embedded in 3D gels as described above, using media M199. At least 10 random 100× fields were examined to assess capillary formation (also called tubulogenesis). The formation of lumen-like structures was visible as early as 24 h. Antibodies were dialyzed in sterile dialysis buffer (25 mM Tris, 175 mM sodium chloride, 2 mM potassium chloride pH 7.4) overnight and passed through a 0.22 μM filter prior to use. The integrity of antibodies was determined by SDS-PAGE. To determine the effects of specific antibodies on pre-formed capillaries, the method of Bayless et al. was used [37,38]. Briefly, at the end of 24 h, mAbs (50 μg/ml) and pAbs (75 μg/ml) were added. Fresh aliquots of antibodies were added every 12 h for a total of 60 h. To quantify the degree of capillary formation, 3D matrices were fixed at 24, 36, 48, 60, and 72 h by aspirating the medium, washing with PBS, and then fixing with 4% glutaraldehyde in PBS, pH 7.4, overnight at 4°C. Matrices were then washed with distilled water and embedded in paraffin according to the manufacturer's instructions (Richard Allen Scientific, Kalamazoo, MI). Serial sections (4 μm) were prepared, dehydrated, stained with acidified eosin, and destained with distilled water. Capillaries were counted and photographed using a Zeiss Axiovert 25C microscope at 100× magnification. Each capillary tubule was surrounded by least 2 to 5 ECs. Capillary formation was defined as the induction of a minimum of 3 separate capillary events within a single field. At least 10 random fields were counted for each sample. Experiments were performed in duplicate, using triplicate wells in each case. Results were expressed as mean ± SEM.

### Statistics

Student's t tests and ANOVA were used to detect significant comparisons as previously described [23].

## Abbreviations

3D, three dimensional; ECs, endothelial cells; bFGF, basic fibroblast growth factor; hr, hour; kDa, kiloDalton; LPP1, lipid phosphate phosphohydrolase-1; LPP3, lipid phosphate phosphohydrolase-3; mAb, monoclonal antibodies; mg, milligram; μg, microgram; pAb, polyclonal antibodies; SDS-PAGE, sodium dodecyl sulphate - Polyacrylamide gel electrophoresis; VEGF, vascular endothelial growth factor;

## Authors' contributions

JOH was responsible for maintaining the monolayer ECs, FACS and protein expression analyses. KKW was responsible for 3D gel preparation, capillary assays, sectioning, staining, data analysis, interpretation, and preparation of manuscript. KKW and JOH were involved in study design, and read and approved the manuscript.
